# Municipal food waste collection strategies in Portugal: A dataset

**DOI:** 10.1016/j.dib.2025.112374

**Published:** 2025-12-10

**Authors:** Diego del Oro Alcalde, Diogo Bugarim, Telmo Coelho, Emília Almeida, Catarina Silva, Luís Cavique, Celia Dias-Ferreira

**Affiliations:** a Department of Siences and Technology (DCeT), Universidade Aberta, Rua da Escola Politécnica, n.º 147 1269-001 Lisboa, Portugal; b LASIGE-FC, Universidade Lisboa, Portugal; c Centre for Functional Ecology, Science for People & the Planet, University of Coimbra, 3000-456 Coimbra, Portugal

**Keywords:** Biowaste, Organic kitchen waste, Door-to-door, Bring-point, Co-collection, Performance indicators, Capture rates, Household collection

## Abstract

The dataset reports an up-to-date overview of the selective biowaste collection with a focus on food waste and organic kitchen waste across 308 municipalities in Portugal, to assess the compliance with the EU Waste Framework Directive that made biowaste collection mandatory from 1st January 2024. Data were collected through a structured survey sent to the totality of the municipalities, complemented by systematic research in secondary official sources such as municipal websites, reports and statistical data. The questionnaire covered aspects such as coverage, collection models (nearby bring points, door-to-door, co-collection), sector-specific deployment (household collection, non-domestic collection), operational characteristics, and performance indicators (capture rates, cost per tonne). The dataset was structured and validated through cross-checking the multiple sources assessed, prioritising direct municipal questionnaire responses. It includes disaggregated data at a municipality level, including detailed information on the characteristics and efficiency of the initiatives, when available. The database allows the cross-comparison across Portuguese regions and potentially with other international systems, in terms of biowaste collection strategies with focus on food waste and organic kitchen waste. Municipalities in Portugal have been carrying out pilot experiences within their territories, but there is no systematic assessment of what has been carried out nor the results obtained. Given the limited available data, this dataset provides a valuable resource for policy design and further research on biowaste management initiatives to further assess their efficiency and adaptability to different municipal realities at a national and even European level.

Specifications Table

**Table:** 

Subject	Earth & Environmental Sciences
Specific subject area	Waste Collection; Biowaste.
Type of data	Tables, Figures, CSV files
Data collection	Data was collected through a structured questionnaire (Microsoft Forms) sent by email to all 308 municipalities in Portugal, obtaining 93 responses. Complementary, data was also collected using a systematic search in secondary official sources such as municipal websites, reports and statistical data.
Data source location	308 Portuguese municipalities, covering both mainland Portugal and the insular territories.
Data accessibility	https://data.mendeley.com/datasets/sv66hbm297/2 Repository name: Dataset in municipal strategies for food waste collection in Portugal. Available in Mendeley Data. Data identification number: doi: 10.17632/sv66hbm297.2 Direct URL to data: https://prod-dcd-datasets-cache-zipfiles.s3.eu-west-1.amazonaws.com/sv66hbm297–2.zip
Related research article	None.

## 1 Value of the Data

• This dataset includes information from 196 food waste collection initiatives implemented across 152 different Portuguese municipalities, based on both questionnaire responses and official secondary sources. The dataset facilitates comparative analyses of different strategies and their relation to several municipal characteristics.
• The data follows a star schema, integrating information on collection types, capture rates, costs and territorial profiles. Official indicators such as population size, area and density have been included, enabling cross-sectoral (domestic vs. non-domestic) and territorial analyses.
• These data support further research on the drivers behind the implementation of new waste collection fluxes, specifically by using the data as explanatory variables for the adoption of food waste collection schemes. In turn, this could further support the analysis of existing barriers and facilitators to the transposition of European waste management policies to the national level.
• The data also supports analysis of the efficiency of selective food waste systems in different territorial contexts. Univariate and multivariate analyses can be performed to identify relations between the municipal characteristics (explanatory variables) and cost-related efficiency parameters, such as the operational cost per tonne of food waste collected and the operational and investment cost per inhabitant.
• The data also supports comparison of different collection model and collection approaches among municipalities, allowing to benchmark collection models based on the efficacy (capture rates) attained.
• This study, along with the presented dataset, contributes to filling a data gap, as there are not any publicly available datasets regarding food waste collection strategies in Portugal. It provides a foundation for further research and policy evaluations on the biowaste management strategies, on a national and international level.

## 2 Background

Biowaste represents 34 % of municipal waste in Europe [1] and 38 % in Portugal [2]. It is composed primarily of food and kitchen waste (60 %) and garden waste (35 %), with the remaining 5 % classified as “others” [3]. Following the EU Waste Framework Directive [4], selective collection of biowaste became mandatory from January 2024, reflecting broader environmental and economic objectives that include efforts to increase recycling rates, reduce greenhouse gas emissions, and support the transition to a circular economy. This legislation represents a challenge for municipalities, which are required to adapt their waste collection systems to comply with the new requirements while simultaneously addressing the environmental and operational demands of the shift toward a circular economy.

Across Europe, approaches to bio-waste collection vary substantially. Some countries or regions collect garden waste but collect little or no food waste, such as most of Denmark, many areas in the Baltic countries and most of France [5]. Others co-collect garden and food waste in the same container, as in the Netherlands, Austria, and Germany [5]. A third group prioritises food waste collection, leaving garden waste as a separate fraction to be collected through civic amenity sites or with specific collection rounds, an approach adopted in Norway, Italy, Wales, Catalonia and the Basque Country [5]. These differences occur not only between countries but also within them resulting in different performances across regions. For example, in Italy in 2023, regional disparities were pronounced, with the Molise Region (in the Center) collecting 86,55 kg/inhabitant whereas Emilia-Romana Region (in the North) reaching 187,84 kg/inhabitant [6].

Performance in biowaste separate collection is generally assessed using three groups of indicators:

(i) effectiveness indicators, the most used being biowaste captured (kg/inhabitant) and capture rates ( % of total biowaste generated that is separately collected);
(ii) quality indicators, primarily contaminant levels ( % of impurities in the biowaste stream);
(iii) efficiency indicators, the most common being operational costs or investment needs per capita or per tonne collected.

Capture rates are highly influenced by the collection models (door-to-door, drop-off points in the streets, civic amenity sites, on‑demand-collection) in combination with the type and configuration of the containers, and the presence of economic incentives, such as pay-as-you-throw schemes [1,7]. Specifically, container type, location and accessibility, including individual kerbside bins, shared bring-points, semi-underground containers, in-home sorting bins and smart/controlled containers, are known to influence participation and capture rates in separate collection [8,9].

The residential structure of municipalities also affects separate collection outcomes, in particular the type of housing namely apartment, multi-family or single-family houses [9]. Detached single-family housing areas are associated with higher participation and lower contamination in biowaste streams [10], whereas multi-residential buildings present logistical and behavioural challenges, often requiring different infrastructuresuch as in-building storage facilities and nearby collection points to make separate collection viable [11].

Geographic and demographic factors, such as population size, population density, degree of urbanisation (rural/urban) further affect collection performance [9]. Research shows that rural areas, characterised by low density and longer average collection distances, typically face higher per capita costs, lower collection efficiency, and a greater likelihood of uncollected waste, compared with urban ones [12].

In Portugal 198 municipalities (out of 278 in mainland) currently implement some form of biowaste separate collection, while 157 municipalities promote source separation, primarily through home composting [2]. Current biowaste capture rates are quite low in most municipalities, specifically between 1–5 % [2]. This data includes both garden waste and food waste, and there is no official dataset exclusively dedicated to food waste collection. Some collection models work well in certain urban typologies (e.g., residential neighbourhoods with detached houses), but not in others (e.g., apartment buildings or mixed-use zones), and although several pilot experiences have been carried out in Portugal, there is no systematic, comprehensive, and up-to-date data on the food waste separate collection systems currently implemented across Portuguese municipalities. This is the gap that the present dataset aims to fill.

## 3 Data Description

**Fig. 1:**
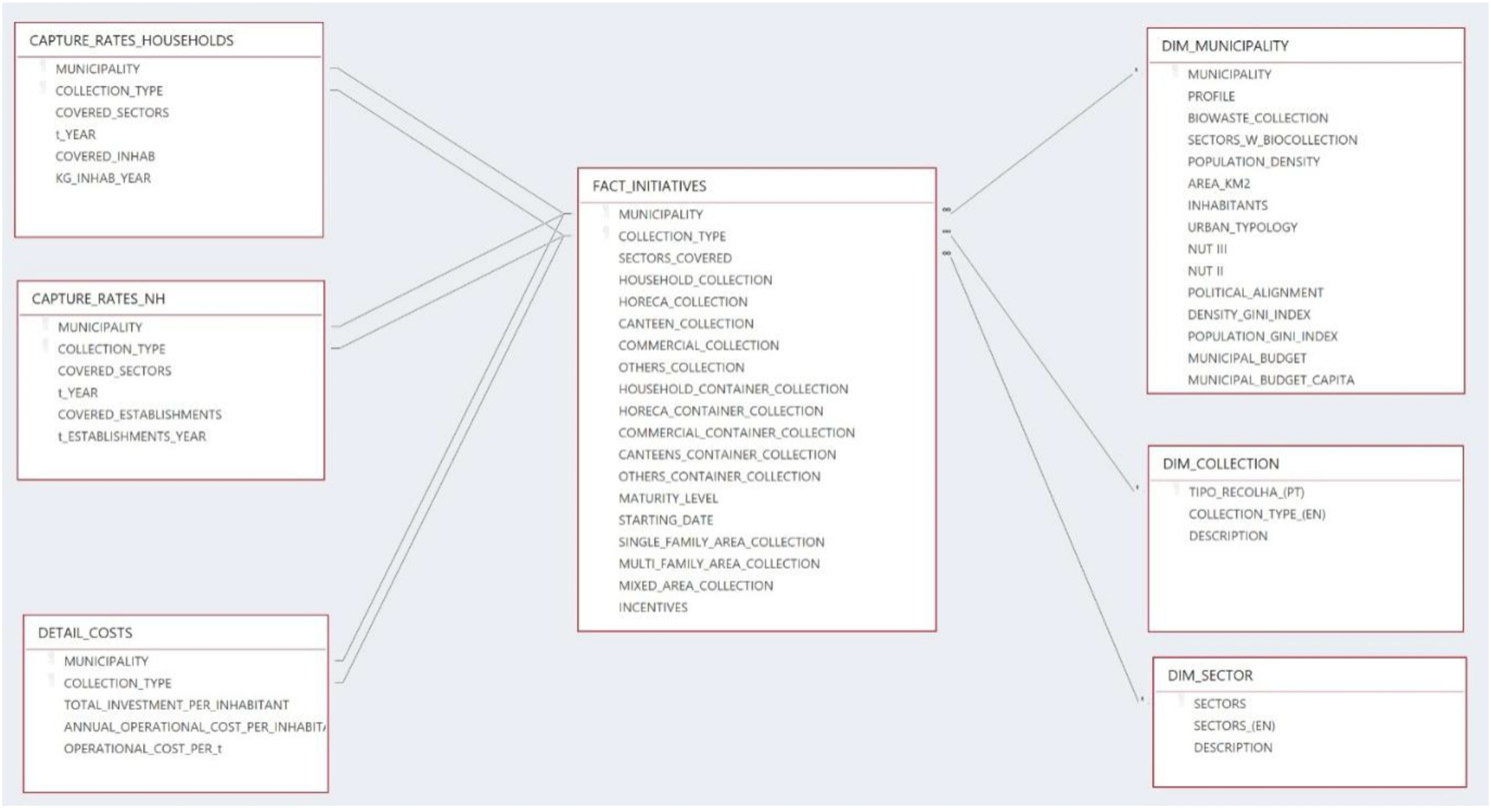
Diagram of the star schema.

The dataset [3] is structured in the form of a star schema (Fig. 1), with one central fact table linked to multiple dimension and detail tables.

The FACT_INITIATIVES table contains information about all 196 food waste collection initiatives implemented by 156 Portuguese municipalities. Each entry describes a specific collection initiative, including information about the covered sectors and operational characteristics. Table 1 lists all the tables in the star database, including a description of the contents.

**Table 1:** Description of the associated star scheme dataset.

Table Name	Type	Contents
FACT_INITIATIVES	Fact Table	Records of food waste collection initiatives, linked to municipality, activity sector, and collection type (see Table 2)
DIM_MUNICIPALITY	Dimension	Information about each municipality based on: Official data: population, area, density, urban typology NUT II, NUT III; political alignment of the municipal executive (2021–2024), Municipal budget. Data directly obtained from the questionnaire: existence of separate collection of food waste at the municipality; economic sectors with foodwaste collection Data calculated by the authors: Gini index for the population distribution; Gini index for the density distribution), profile of the municipality regarding foodwaste collection (classification based on the data obtained in the questionnaire); Municipal budget per capita
DIM_COLLECTION	Dimension	Description of the type and features of the food waste collection systems
DIM_SECTOR	Dimension	Description of the target sectors of the initiatives (domestic, non-domestic, mixed)
CAPTURE_RATES_HOUSEHOLDS	Detail (1:1)	Captured rates, in kg per inhabitant per year, in households, including some services in the city fabric
CAPTURE_RATES_ND	Detail (1:1)	Capture rate, in tonnes per establishment in exclusive non-household collections, if available
DETAIL_COSTS	Detail (1:1)	Financial information related to the initiative (costs/year, €/ton), if available

The dataset is uploaded in the repository Mendeley titled “Dataset in municipal strategies for food waste collection in Portugal” [13], which contains the star schema dataset in 7 CSVs and the questionnaire in PDF format, which provides context for the collected variables.

## 4 Experimental Design, Materials and Methods

### 4.1 Data selection

The selection of data to be included in the dataset was guided by the literature reviewed and by the professional experience of the authors, reflecting theoretical, empirical and policy relevant considerations related to the implementation and performance of separate food waste collection systems. The variables fall into two main categories: (i) geographic, socio-economic, and institutional characteristics of municipalities, and (ii) variables directly related to the design and operation of separate collection schemes.

(i) Geographic, socio-economic, and institutional characteristics

Separate waste collection of food waste is under the responsibility of the municipality and therefore the municipal context plays a decisive role in shaping waste-management strategies and the feasibility of different collection models. For this reason, the dataset includes several structural variables. Population, area and population density are classical descriptors of municipal morphology and settlement patterns. To distinguish between rural and urban we have used the official variable “urban typology” of the municipality, which classifies municipalities in three levels: predominantly rural, moderatly urban and predominantly urban).

Gini-based indexes were used to quantify the degree to which residents are unevenly spread across spatial units (census tracts). A Gini value close to 0 indicates a relatively uniform distribution of population across the municipality, whereas a value approaching 1 reflects strong concentration in a limited area. To account for regional differences (as highlighted in the background section), the NUTII and NUT III were included in the dataset. The political alignment and municipal budget available in each municipality might also be related to the waste collection options, so these variables were also included in the dataset.

(ii) Variables related to separate waste-collection systems

**Fig. 2:**
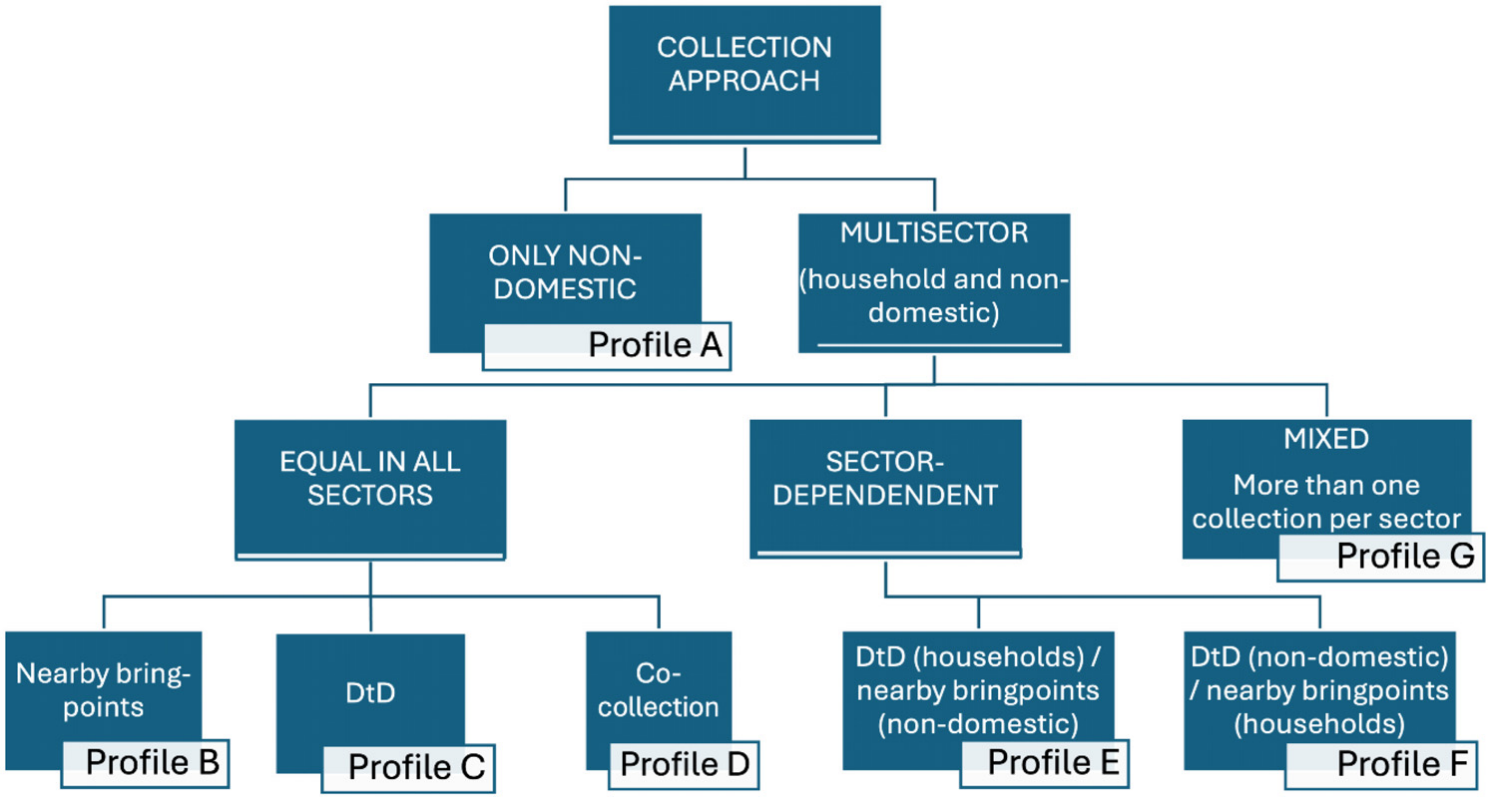
Diagram explanation of the municipality profile classification.

To characterise the food-waste collection initiatives themselves (Fig. 2), the dataset includes variables reflecting the design choices, target groups, and outcomes of the systems.

Captured amounts of food waste, contaminant levels and operational costs per unit waste or per inhabitant constitute key performance indicators, reflecting both user participation and the operational effectiveness of the system. Including these variables allows for the evaluation of outcomes in relation to contextual factors and collection design choices, such as the collection model used (nearby bring-points, door-to-door, and co-colection) and types of containers (individual bins, shared containers, smart bins, etc.).

Housing type (detached houses vs. multi-residential buildings) is essential because the feasibility and performance of collection models, particularly door-to-door, vary substantially with the built environment and dwelling typologies. Economic sectors targeted (e.g., households, commerce, services) capture the scope of the initiative, acknowledging that coverage and performance differ substantially depending on whether the system targets only domestic producers or includes commercial and institutional generators.

## 5 Data Collection

### 5.1 Survey

A structured questionnaire was designed to assess the municipal initiatives in place until 2025 for the selective collection of biowaste in Portugal. Before conducting the extensive survey, a pre-trial was conducted (one municipality was asked to answer) to ensure linguistic clarity and relevance of questions. The results of the pre-trial were used to make adjustments to the wording of the questions.

The questionnaire was made available in Microsoft Forms and was distributed via email to the waste management officials responsible for biowaste collection in all 308 municipalities of Portugal. The questionnaire was sent on 14/03/2025. After four weeks, it was resent to those municipalities that had not yet responded. Answers were received up to 16/06/2025.

The questionnaire’s had an introductory section addressing the name of the municipality, whether the municipality had separate collection of food and kitchen waste and which alternatives were available for biowaste management (e.g. composting). In case the municipality did not have separate collection of fod waste the questionaire would terminate after the introductory section. Those municipalities that had separate collection moved forward to the main part of the questionnaire, which was structured around three predominant collection models: nearby bring-points, door-to-door, and co-collection. Each collection model was addressed in an independent section, containing up to 21 questions which were structured around four topics:

1. Activity sector and access to waste containers: Municipalities were asked to identify the activity sectors covered by each collection model applied on their territory, including households, HoReCa (Hotels-Restaurants-Cafeterias), food retail and canteens, specifying the type of access to the biowaste containers (free or controlled) for each urban typology. The implementation dates of the initiatives were also questioned.
2. Waste producers and captured quantities: The questions from this section addressed the number of inhabitants, households or establishments included in the biowaste collection initiatives and the captured quantities in each case (tonnes/inhabitants/year, tons per year, cumulative tonnes or other indicators).
3. Changes in mixed/residual waste collection after the biowaste initiative started: This set of questions addressed the potential changes in the regular waste collection system after the start of the selective biowaste collection. It addressed changes in frequency, collection circuits or volume of waste containers, including comparative answers before and after the implementation of the separate collection initiative.
4. Economic Details: Municipalities were asked to detail the investments and operational costs associated with the implementation of the biowaste collection initiative. Additionally, the presence of economic incentives, such as Pay-as-you-throw (PAYT), was assessed.

### 5.2 Website research

Official sources (municipal websites and social media pages, waste management operators’ reports) and reliable online news articles were systematically reviewed to collect additional data regarding biowaste collection initiatives across Portugal. In parallel, a documentary *corpus* was created and archived, comprising prints of all webpages and documents consulted. These records were organised by municipality and served, at a later stage, as a reference to validate and trace the origin of the different entries in the database.

### 5.3 Data processing

Data from survey answers and secondary official research sources were consolidated into an XLSX file, structured as a database. A cross-check process was conducted for all entries, flagging missing data as “-”, and resolving discrepancies by prioritising information directly obtained from the municipalities over secondary sources, and further contacting municipal waste managers in cases of abnormal values. Obtaining a total of 24 different variables, as in Table 2*.*

**Table 2:** Description of variables on municipal biowaste initiatives.

Variable Name	Description	Data Type	Source	Value Range / Units
MUNICIPALITY	Full name of the Portuguese municipality	Geographic (Text)	NGIS	N/A
COLLECTION_ TYPE	Type of biowaste collection model implemented in the initiative	Categorical (Nominal)	Quest & O.s.s	Nearby bring points, Door-to-door, Co-collection
COVERED_ SECTORS	Activity sectors covered by the initiative	Categorical (Nominal)	Quest & O.s.s	Household, Non-household, Mixed
HOUSEHOLD_ COLLECTION	Whether the household sector is included	Binary	Quest & O.s.s	0 = No or unknown, 1 = Yes
HORECA_ COLLECTION	Whether the HORECA (hotels- restaurants-cafeterias) sector is included	Binary	Quest & O.s.s	0 = No or unknown, 1 = Yes
CANTEENS_ COLLECTION	Whether institutional canteens (schools, hospitals, etc.) are included	Binary	Quest & O.s.s	0 = No or unknown, 1 = Yes
COMMERCIAL_COLLECTION	Whether food retail and markets are included	Binary	Quest & O.s.s	0 = No or unknown, 1 = Yes
OTHER_COLLECTION	Whether other sectors (not listed above) are included	Binary	Quest & O.s.s	0 = No or unknown, 1 = Yes
PROFILE	Classification of the municipal approach to selective biowaste collection	Encoded Classification	Author-defined	As defined in Fig. 2.
BIOWASTE_ COLLECTION	Types of collection models present in the municipality	Encoded (Integer)	Quest & O.s.s	1-Door-to-door 2- Bringpoints 3-Co-collection 4-Door-to-door & Bringpoints 5-Bringpoints & Co-collection 6-Door-to-door & Co-collection 7-All 8-None.
SECTORS_ W_BIOCOLLECTION	Sectors covered by biowaste collection in the municipality	Encoded (Integer)	Quest & O.s.s	1 = Household, 2 = Non-domestic, 3 = Both, 4 = None
*_CONTAINER_COLLECTION	Type of collection per sector (e.g., household, HORECA, etc.)	Encoded (Integer)	Quest & O.s.s	0–7 (see legend below)
MATURITY_ LEVEL	Maturity level of the biowaste collection system	Ordinal Scale	Quest & O.s.s	1 = Incipient (<3 month); 2 = Developing (3–12 months); 3 = Mature (>1 yr)
STARTING_ DATE	Year or date of implementation	Date / Year (Text)	Quest & O.s.s	e.g., 2023, 01/04/2025
*_AREA_COLLECTION	Type of collection in single-family, multi-family, or mixed areas	Encoded (Integer)	Quest & O.s.s	0–7 (see legend below)
INCENTIVES	Whether incentive mechanisms (e.g., PAYT) are present	Binary	Quest & O.s.s	0 = No or unknown; 1 = Yes
t_YEAR	Annual tonnage of biowaste collected	Numeric (Continuous)	Quest & O.s.s	Tonnes/year
COVERED_ INHAB	Number of inhabitants covered by the system	Numeric (Integer)	Quest & O.s.s	Number of inhabitants
COVERED_ ESTABLISHMENTS	Number of establishments covered	Numeric (Integer)	Quest & O.s.s	Number of establishments
ANNUAL_ COSTS	Annual operational costs of the system	Numeric (Continuous)	Quest.	Euros (€)
KG_INHAB_ YEAR	Biowaste captured per capita in kilograms per year	Numeric (Continuous)	Derived	kg/inhabitant/year
t_ESTABLISHMENT_YEAR	Biowaste captured per establishment per year	Numeric (Continuous)	Derived	tonnes/establishment/year
COST_PER_t	Operational cost per tonne of collected biowaste	Numeric (Continuous)	Quest.	€/tonne
COST_PER_INHAB	Operational cost per capita	Numeric (Continuous)	Quest.	€/inhabitant

Legend for Encoded Fields: 1. (*_CONTAINER_COLLECTION and *_AREA_COLLECTION):

0 = No selective collection / Not applicable; 1 = Nearby bring point (free access); 2 = Nearby bring point (controlled access); 3 = Nearby bring point (mixed access); 4 = Door-to-door with containers; 5 = Door-to-door with bags; 6 = Door-to-door with containers and bags; 7 = Co-collection.

2. Source: Quest = Questionnaire; O.s.s. = Official secondary sources; NGIS = National Geographic Information System

## Limitations

The main limitation of the former study is the scarcity of information available regarding biowaste collection, specifically quantitative parameters regarding capture rates and costs. With the available data, correctly assessing the efficiency of the different systems becomes challenging. Additionally, the questionnaire was answered by nearly 100 municipalities, which represent one-third of the potential respondents. The data for the remaining municipalities had to be taken from online sources, which may not always provide accurate or up-to-date information. While the launch of a municipal initiative for food-waste collection is very likely to be reported in news outlets, such as municipal websites, online newspapers, and the municipality’s social-media accounts, the subsequent evolution of the initiative is much less consistently reported. Data on capture rates, performance over time, and operational costs are often missing or might be outdated.

## Ethics Statement

The current study does not involve human subjects, animal experiments or data collection from social media sources. The authors confirm that they have read and followed the ethical requirements for publication in Data in Brief.

## CRediT Author Statement

**Catarina Silva:** Investigation; **Celia Dias-Ferreira:** Conceptualization, Methodology, Formal analysis, Investigation, Writing - Original Draft, Writing - Review & Editing, Supervision, Funding acquisition; **Diego del Oro Alcalde:** Methodology, Validation, Formal analysis, Investigation, Data curation, Writing - Original Draft, Visualization. **Diogo Bugarim:** Methodology, Validation, Investigation, Writing - Review & Editing, Supervision; **Emilia Almeida:** Investigation; **Luís Cavique:** Formal analysis, Writing - Review & Editing, Visualization; **Telmo Coelho:** Investigation.

## Data Availability

Mendeley DataDataset in municipal strategies for food waste collection in Portugal (Original data).
